# Development of Perineuronal Nets during Ontogeny Correlates with Sensorimotor Vocal Learning in Canaries

**DOI:** 10.1523/ENEURO.0361-19.2020

**Published:** 2020-03-26

**Authors:** Gilles Cornez, Clémentine Collignon, Wendt Müller, Charlotte A. Cornil, Gregory F. Ball, Jacques Balthazart

**Affiliations:** 1 Behavioral Neuroendocrinology Lab, GIGA Neurosciences, University of Liege, Liege 4000, Belgium; 2 Behavioural Ecology and Ecophysiology Research group, University of Antwerp, Antwerp 2000, Belgium; 3 Department of Psychology, University of Maryland, College Park, 20742, MD

**Keywords:** parvalbumin, sensorimotor learning, *Serinus canaria*, song learning, song system, testosterone

## Abstract

Songbirds are a powerful model to study vocal learning given that aspects of the underlying behavioral and neurobiological mechanisms are analogous in many ways to mechanisms involved in speech learning. Perineuronal nets (PNNs) represent one of the mechanisms controlling the closing of sensitive periods for vocal learning in the songbird brain. In zebra finches, PNN develop around parvalbumin (PV)-expressing interneurons in selected song control nuclei during ontogeny and their development is delayed if juveniles are deprived of a tutor. However, song learning in zebra finches takes place during a relatively short period of development, and it is difficult to determine whether PNN development correlates with the end of the sensory or the sensorimotor learning period. Canaries have a longer period of sensorimotor vocal learning, spanning over their first year of life so that it should be easier to test whether PNN development correlates with the end of sensory or sensorimotor vocal learning. Here, we quantified PNN around PV-interneurons in the brain of male canaries from hatching until the first breeding season and analyzed in parallel the development of their song. PNN development around PV-interneurons specifically took place and their number reached its maximum around the end of the sensorimotor learning stage, well after the end of sensory vocal learning, and correlated with song development. This suggests that PNN are specifically involved in the termination of the sensitive period for sensorimotor vocal learning.

## Abstract (precis)

**Figure:**
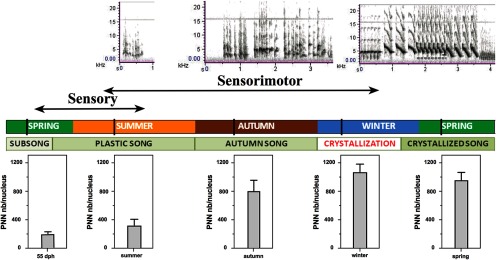

## Significance Statement

Perineuronal nets (PNN) are accumulations of components of the extracellular matrix that form usually around parvalbumin (PV)-expressing inhibitory neurons. PNN have been associated with various forms of experience-dependent or activity-dependent learning in mammals where they appear to control the end of sensitive periods for learning. It was recently demonstrated that PNN are associated with vocal learning in juveniles and adults of several species of songbirds, but the specific aspect of the learning process they control has not been formally identified. We demonstrate here that during ontogeny in male canaries, PNN develop essentially during the sensorimotor phase of song learning, which suggests that they represent part of the neuronal mechanisms underlying song crystallization.

## Introduction

Songbirds represent a canonical model system to study vocal learning (Moorman and Bolhuis, 2013). Songbirds learn their song through social interactions during development based on conspecific song usually provided by a tutor though tape recordings are effective in some species (Marler, 1970; Peters and Marler, 1977; Waser and Marler, 1977; Baptista and Gaunt, 1997; Goldstein et al., 2003; Thorpe, 2008).

Song learning during development can be divided into a sensory phase during which the tutor song is memorized and a sensorimotor phase during which song production is progressively refined to match the memorized song template (Marler, 1991; Brenowitz et al., 1997; Williams, 2004). Some songbird species, called closed-ended learners, never change their song after a limited period of learning during development, whereas other species, called open-ended learners, can in addition modify their song during adulthood on a seasonal basis (Brainard and Doupe, 2002; Brenowitz and Beecher, 2005).

Studies of songbirds provided significant insights into the neurobiological processes involved in vocal learning (Brenowitz and Beecher, 2005; Pfenning et al., 2014). Songbirds possess a set of interconnected brain nuclei, called the song control system, that specifically underlies song learning and production (Mooney, 2009a; Nottebohm, 2005) and is analogous to the brain nuclei involved in language learning and production in humans (Pfenning et al., 2014). Nucleus HVC (used as a proper name) is connected to RA (robust nucleus of the arcopallium), a premotor nucleus, and together these nuclei control the motor aspects of song production (Mooney, 2009b). Additionally, HVC is indirectly connected to RA via area X of the striatum, DLM (medial part of the dorsolateral nucleus of the thalamus), and lMAN (the lateral magnocellular nucleus of the anterior nidopallium). This circuit is involved in song learning and also in the control of adult song variability (Brainard, 2004).

Developmental song learning occurs during a sensitive period of neural plasticity associated with neurogenesis (Nordeen and Nordeen, 1988; Kirn and DeVoogd, 1989; Bottjer and Arnold, 1997) and synaptic pruning (Miller-Sims and Bottjer, 2012) in the song control system. Additionally it was recently suggested that perineuronal nets (PNNs) could play an important role in the regulation of sensitive periods for vocal learning in a closed-ended learner species, the zebra finch, *Taeniopygia guttata* (Balmer et al., 2009; Cornez et al., 2017b, 2018). PNN are aggregations of chondroitin sulfate proteoglycans, tenascin R, hyaluronic acid and binding proteins that form a scaffold mainly around fast spiking interneurons expressing parvalbumin (PV; Deepa et al., 2006; Wang and Fawcett, 2012). They stabilize synaptic connectivity by preventing establishment of new synaptic contacts (Karetko and Skangiel-Kramska, 2009) and increase the fast spiking activity of PV interneurons supporting the precise timing of neural inhibition (Balmer, 2016). PNN are thus assumed to play an important role in the closing of sensitive periods for sensory learning (Hensch, 2004; Werker and Hensch, 2015). Adult male zebra finches have more PNN in HVC than juveniles (Balmer et al., 2009) and their development around PV-interneurons is inhibited by acoustic isolation (Balmer et al., 2009). We previously reported that PNN develop in the song control system of male zebra finches between the end of the sensory and the end of the sensorimotor phase of song learning (Cornez et al., 2018) but the overlap between these two phases (Brainard and Doupe, 2002; Williams, 2004) makes it difficult to link PNN to a specific process. In contrast, song learning in canaries takes place over an extended period during ontogeny that only ends during the winter or early spring following hatching in the previous summer (Brainard and Doupe, 2002; Leitner et al., 2015). Sensory learning starts after birds fledge (probably between 25 and 35 dph) and ends between the summer and early fall. Since adult birds stop singing in the summer, juveniles presumably stop acquiring their song template at that time. The sensorimotor learning phase, which roughly begins in the middle of the sensory phase, however extends until the winter or even the early spring, so that it is easier to separate the two processes even if they partly overlap. If PNN close the sensory period, their development should be completed in the summer but if they relate to the sensorimotor aspect of song learning, they should develop later during the year. Here, we tested this idea by analyzing during ontogeny the progress of song learning and PNN development in the song control system in groups of male canaries that hatched at the same time but were recorded and sampled for PNN development at key points during their first year of life.

## Materials and Methods

### Subjects

Juvenile male canaries of the Fife fancy breed (*n* = 49) arrived at the GIGA Neurosciences, University of Liege, on May 19th 2016 at around 55 days post-hatch, dph (range 45–60 dph). All birds originated from a large pedigreed, outbred canary population that is maintained in the animal facilities of Behavioral Ecology and Ecophysiology Research group at the University of Antwerp, Belgium. In this particular case, all birds were raised in the context of an artificial selection experiment for high and low begging behavior, and belonged to the third or fourth generation (Fresneau, 2017). These birds thus had slightly different but known background and this information was used to evenly distribute birds across the experimental groups (see next section). In Antwerp, birds were kept in cages along with their parents from hatching until 25 dph old. They were then moved to collective cages of 10 fledglings with one adult male until they were transferred to the University of Liège at ∼55 dph. All birds were molecularly sexed (PCR) before being selected for this experiment (Griffiths et al., 1998). From birth until transfer, birds were kept in an indoor animal facility under a photoperiod that corresponds to the natural photoperiod at the latitude of Belgium. On day of arrival at the GIGA Neurosciences, the photoperiod was set at 16 h of light and 8 h of darkness (16L:8D). Five additional adult males (more than two years old) were also brought to the University of Liege on the same day from the University of Antwerp and served as tutors during the entire experiment. Upon arrival at the GIGA Neurosciences, all juvenile birds were housed in a collective indoor aviary, whereas adult tutors were kept in a collective cage facing the aviary in the same room. Since the period of sensory learning only starts after birds fledge, probably between 25 and 35 dph (Brainard and Doupe, 2002; Leitner et al., 2015), all subjects of the present experiment were exposed largely to the same tutoring regimen. Food and water were always provided *ad libitum*. Cuttlebones, anise-scented sand, perches and baths were provided as environmental enrichment. Egg food was provided once a week. During recording sessions, birds were kept in individual cages within a sound-attenuated chamber which allowed obtaining high-quality recordings of songs from individually identified subjects. Food, water and enrichment were provided the same way as in the aviary. All experimental procedures complied with Belgian laws concerning the Protection and Welfare of Animals and the Protection of Experimental Animals, and experimental protocols were approved by the Ethics Committee for the Use of Animals at the University of Liège (Protocol 1739).

### Experimental design

During the entire experiment, the photoperiod was adjusted on the 20th of each month to match the outside natural photoperiod at the latitude of Belgium (16L:8D on arrival in the lab). The experimental birds were continuously exposed to the five adult male tutors to ensure that sensory learning was not interrupted. Juvenile male canaries were allocated to five experimental groups to be studied in different seasons and stages of song learning.

Canaries like other songbird species first produce unstructured vocalizations similar to the babbling of human infants: the subsong. This is followed by a period of plastic song that resembles the typical adult song with the appearance of syllables, but these syllables are still poorly structured and quite variable between successive renditions. In adult birds, song has a precisely defined structure with identifiable syllables. This is the crystallized song which is used by adult males to attract females and repel competing males (Doupe and Kuhl, 1999; Brainard and Doupe, 2002; Williams, 2004).

**Figure 1.**
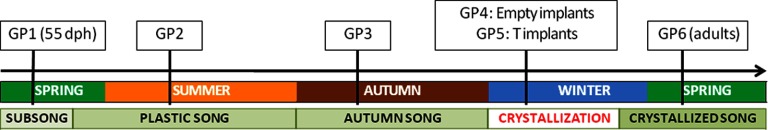
Timeline representing the first year of life of canaries from hatching until the first breeding season. Sensorimotor learning stages and seasons appear below (autumn song corresponds to the latest period of plastic song). Vertical lines indicate the periods when brains were collected for each group.

In canaries sensory learning starts at fledging (around 25–35 dph) and ends sometimes during the summer when adults stop singing. Young birds are at that time between 50 and 100 d old), depending on whether they hatched early or late in the season (Leitner et al., 2015). In contrast, sensorimotor learning starts around 60 dph and extends until the first breeding season when birds are around one year old (Brainard and Doupe, 2002; Leitner et al., 2015). Increases of PNN expression occurring after the summer would consequently be associated with sensorimotor learning rather than with the sensory period. Therefore, singing behavior was recorded and brains were collected at five different time points between 55 dph until the onset of the first breeding season in early spring. Additionally, a subset of birds was treated with testosterone (T) during the winter to test whether premature crystallization of the song would be associated with enhanced PNN expression. At the pre-determined stage, all males from one group were transferred to individual sound-attenuated chambers to record their singing behavior during four weeks before their brain was collected. Each experimental group thus corresponds to a specific developmental age as well as to the corresponding season, which matches the natural conditions of canary song development during the first year of life (see Fig. 1).

Song in the first group (55 dph, *n* = 10) was not recorded before brain collection because these birds had not started singing yet. Their brains were collected on May 24 (mean age of this group: 55 dph, range 50–58 dph). The second group (summer, *n* = 8) was recorded in early summer, their brains were collected on July 19 (mean age: 118 dph, range 116–122 dph) when the increasing photoperiod was set at 16.5L:7.5D. The third group (autumn, *n* = 8) was recorded in early autumn and their brains were collected on October 24 (mean age: 215 dph, range 212–217 dph) under a decreasing photoperiod of 12.3L:11.7D. The fourth group was subdivided into two subgroups that received a 10 mm long SILASTIC implant filled with crystalline T or left empty as control (ctrl) to study the potential effect of T-induced premature crystallization on the expression of PV and PNN (winter ctrl, *n* = 7; winter+T, *n* = 8). T has previously been shown to activate singing activity in adult male and female canaries (Madison et al., 2015; Cornez et al., 2017a; Vellema et al., 2019). These two sub-groups were recorded in early winter and their brains were collected on February third (mean age: 317 dph, range 314–320 dph) under a photoperiod of 8L:16D. The fifth group (spring, *n* = 7) was recorded in early spring of the subsequent year, at the onset of the breeding season, and their brains were collected on April 19 (mean age: 392 dph, range 389–393 dph) under an increasing photoperiod of 12.2L:11.8D.

Two days before the beginning of the recording sessions, birds from the corresponding group were caught with a net in the aviary and transferred in a collective cage during the afternoon. This procedure was performed to allow a faster catching of each individual on the following day during which we collected a blood sample and transferred each bird into his individual recording chamber. On the day of brain collection, we measured the body weight, the syrinx weight, and the mean testes weight of each subject. One bird from the spring group died before the onset of recordings, which reduced the final sample size to 48 subjects.

### Implant insertion

In birds of the winter group, implants were inserted subcutaneously under isoflurane gas anesthesia during the late morning 4 d after the start of the recording session. A 10-mm-long SILASTIC implant (Dow Corning reference no. 508–004; inner diameter, 0.76 mm and outer diameter, 1.65 mm) filled with either crystalline T (Fluka Analytical, Sigma-Aldrich) or left empty as a control was inserted subcutaneously in the back of the birds. Before implantation, each implant was carefully checked under a stereo-microscope to make sure it was completely sealed and implants were incubated in 0.9% NaCl at 37°C overnight before being inserted. A small hole was made in the skin in the apterium located at the back of the neck, the implant was inserted and the hole was sutured with a 5–0 coated Vicryl thread. This procedure took <5 min. Birds were then allowed to recover in an individual cage under a warm lamp and they all recovered (moving and perching normally) within 10 min. They were then transferred back to the recording chamber until brains were collected. Although birds from the other age groups were not exposed to anesthesia and implant surgery, it is unlikely that this brief minor procedure interfered with any of the measures presented here.

### T enzyme immunoassay (EIA)

To study the sexual development of the males during the course of the experiment, blood (50–150 μl) was collected from the wing vein of each bird on the day before the beginning of the recording sessions and on the day of brain collection. Blood collection was always performed within 3 min after catching the birds in their collective cage during the morning, within 1.5 h after the lights went on. For the winter group, the order of collection of blood samples was counterbalanced across implant conditions. Blood was collected into Na-heparinized micropipettes and any further blood flow was stopped by pressing cotton on the vein puncture after a maximum of 150 μl was collected. An additional blood sample was taken for the winter group 15 d after the pre-recording sample (10 d after implant insertion) with a maximum of 100 μl to explore the changes in time of T concentration following implant insertion. Blood was directly centrifuged at 9000 × *g* for 9 min, and the supernatant plasma was stored at −80°C until further use.

Plasma (10 μl) from each sample was diluted in 150 μl of ultra-pure water. Three additional samples were spiked with 20,000 CPM of tritiated-T (PerkinElmer) to estimate the recovery after extraction. All samples were extracted twice with 2 ml of dichloromethane. The organic phase was eluted into clean tubes, dried with nitrogen gas and stored at −20°C until further use. Average recovery rate was 71.7% (68.45–81.25%).

Extracted samples were re-suspended in 400-μl EIA buffer by vortexing for 30 s and shaking for 120 min at 1350 rpm. A fraction (50 μl) of the re-suspended samples was placed in each assay well. Samples (*n* = 106) were assayed in triplicate for T concentration using a Cayman Chemicals T EIA kit following manufacturer’s instructions using five assay plates. The minimum and maximum detection limits of the EIA, as determined by the lowest and highest concentration detected within the standard curves, were 0.13 and 25.47 pg/well, respectively. Concentration of two samples was below this detection limit; they came from 55-dph birds at the time of brain collection and their T level, as extrapolated, was respectively 0.11 and 0.15 ng/ml. The intraassay coefficient of variation varied between 2.5 and 4.1% (mean = 2.9%) and the interplate coefficient of variation ranged from 7.0% to 27.0% (mean = 16.2%).

### Song analysis

When birds were individually housed in sound-attenuated chambers, their singing behavior was recorded every day during two consecutive hours starting directly after lights went on. This procedure was followed for a total duration of four weeks. Sounds from all chambers were acquired simultaneously via custom-made microphones (microphone from Projects Unlimited/Audio Products Division, amplifier from Maxim Integrated) through an Allen & Heath ICE-16 multichannel recorder connected to a computer. The sound files were 16-bit acquired at a frequency of 44,100 Hz which translates to a frequency range of 0–22,050 Hz and saved as 1 min .wav files sequences using Raven Pro v1.4 software (Bioacoustics Research Program 2011; Raven Pro: Interactive Sound Analysis Software, version 1.4; The Cornell Lab of Ornithology).

The sound analyses were performed with the same software. The daily 2-h song recordings were first reassembled for each channel corresponding to each experimental bird. Spectrogram views of these files were constructed with a direct Fourier transform (DFT) size of 256 samples (172 Hz per sample) and a temporal frame overlap of 50% with a hop size of 128 samples. These parameters were automatically determined by the software to provide an optimized frequency/time resolution for the spectrographic analysis and were identical for all recordings analyzed in the study.

The first hour of recordings obtained 2 d before brain collection was analyzed in detail for each bird. One hour of recording was sufficient to obtain at least 240 s of songs for each bird, the duration of vocalizations necessary and sufficient to identify the complete repertoire of the canary (Halle et al., 2003). Analysis of this duration of recording also provided estimates of various song parameters associated with a low degree of variation suggesting that these measures represent reliable estimates of an individual’s song structure.

Vocalizations were considered as distinct songs if they lasted at least 0.5 s and if they were separated by a gap of minimum 0.5 s. Some previous studies used a minimum song duration of 1 s (Leitner et al., 2001; Alward et al., 2013, 2017), but this criterion cannot be applied for juvenile canaries that barely produce songs. The minimum sound duration to be considered as a song was then diminished and calls that are isolated single-frequency vocalizations were visually excluded from the analyses. All songs corresponding to the criteria were manually selected through the entire 1-h-long recording and counted (song numbers; see results in Fig. 3). The duration of each song was provided by the software and these durations were averaged for each bird and averaged each day. These measures were also summed to provide the total duration (in seconds) of singing during 1 h that was then divided by 3600 to obtain the percentage of time spent singing (% time singing).

Each individual song as a whole was also processed through the automated sound analysis of the Raven software. The additional measurements obtained in this way characterized the song “loudness” [average and maximum power (dB), root mean square (RMS), and maximum amplitude (U)], the energy distribution across frequencies [5%, first quartile, center, third quartile, and 95% frequencies (Hz)], the bandwidth (Hz) of this energy distribution between the first and third quartile [interquartile (IQR) bandwidth) and between 5% and 95% (90% bandwidth), the frequency at which the maximum power occurred [maximum frequency (Hz)] and the average entropy (bits; for more details see the software user manual at https://www.raven.com/pages/user-manuals). These derived measures refer to entire songs not to individual syllables. Specifically, the measures of frequencies relate to the distribution of the energy across the entire song. For example, the center frequency indicates the frequency that divides, on average, the distribution of energy in half over the entire song. The entropy associated with the distribution of power across frequencies was measured at each sampling time point across the entire song and averaged to provide a single measure for each song.

Parameters of recordings and spectrogram DFT transform parameters (see first paragraph of this section) led to a time resolution of 5.8 ms and a frequency resolution of 86.1 Hz. For the measure of entropy specifically, this means that for each 5.8-ms frame, the energy distribution across all 86.1-Hz blocks was calculated in the total 22,000-Hz range. If all sound energy occurred in one frame, entropy was equal to zero. As whole songs were selected for these analyses, periods of silence between syllables and trills are included in the analysis, but this only represents a negligible part of the selection.

These measures were then averaged for the entire recording of the day for each bird and they provided a measure of disorder within the energy distribution. Lower song entropy is associated with a higher precision in producing sound energy at specific frequencies, which represents one of the many features of the adult stereotyped song as compared with plastic song. In castrated male canaries, four weeks of exposure to T progressively improved song quality as reflected by a longer duration, higher energy and decreased entropy measured at the level on entire songs (Cornez et al., 2017a). Similarly, T has been shown to promote development in female canaries of more stable male-like songs associated with lower entropy as measured at the level of individual syllables (Vellema et al., 2019). All measures were averaged for each bird and each day.

**Figure 4.**
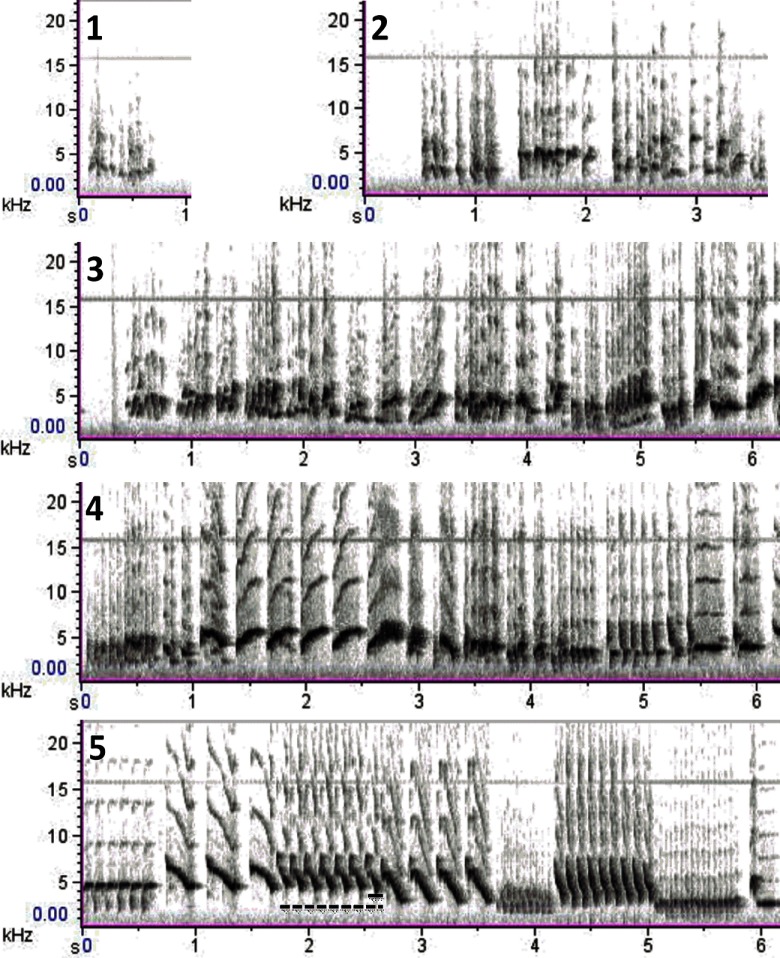
Representative spectrograms illustrating each stage of song development based on the criteria used to calculate the song developmental score (Table 1). The different panels illustrate subsong (**1**), advanced subsong (**2**), plastic song (**3**), advanced plastic song (**4**), and crystallized song (**5**). In the crystallized song, the dotted line indicates a phrase and the full line indicates a syllable.

Additionally, we attributed a semi-quantitative developmental score ranging from 1 to 5 to each selected song that characterized the level of song development from subsong (1), through advanced subsong (2), plastic song (3), advanced plastic song (4) to crystallized song (5). Briefly, the score was assigned following spectrogram inspection based on multiple qualitative criteria including the possibility of identifying individual syllables, the presence of a song structure typical of the canary song including different phrases that are repetitions of a same syllable, the sharp representation of syllables in the sonograms indicating the presence of crystallized song syllables and the general accuracy of syllable repetition in terms of frequency and time (see detailed criteria in Table 1 and spectrographic illustrations of the song development in Fig. 4). For these evaluations the rater was not blind to the age of the birds because the song file name contained the date of recording but the rater was blind to whether the males had been treated or not with T in the winter samples. We suggest that all these scores are nevertheless reliable because: (1) differences between ages are large and partly based on purely objective criteria (e.g., song duration), (2) the blind evaluation of the two winter groups reliably identified differences of a smaller magnitude. For each bird, the score of all songs was averaged to obtain a mean developmental score. A similar measure of song development was previously used to study the effect of T on song development in juvenile song sparrows (Templeton et al., 2012). We used here a similar development scale but the criteria corresponding to each grade were adapted to the specificity of the canary song.

**Table 1:** Criteria used to assign a qualitative developmental score to songs produced by first year male canaries

Developmental score criteria	Song duration	Song structure^1^	Syllable structure^2^	Repetition accuracy^3^
Early subsong, score = 1	>0.5 s	No	Not clearly discernable	No
Advanced subsong, score = 2	>1.0 s	No	Not clearly discernable	No
Plastic song, score = 3	>0.5 s	Structure starts to appear	Not clearly discernable	No
Advanced plastic song, score = 4	>0.5 s	Apparent structure	Some syllables discernable	Some phrases accurately repeated
Crystallized song, score = 5	>0.5 s	Apparent structure	All syllables discernable	All phrases accurately repeated

The developmental score is based on the qualitative evaluation of the song structure, syllable utterance, and syllable repetition accuracy compared with an adult male canary during the breeding season.

1 Typical structure of an adult male canary contains different phrases made of syllable repetitions.

2 Similar to spectrogram view from an adult song.

3 Without visible changes in time-frequency contours between successive renditions within the spectrogram.

### Tissue collection and immunohistochemistry

After the recording sessions, subjects were weighed, their cloacal protuberance was measured, a blood sample was taken from the wing vein and birds were then anaesthetized with Nembutal (0.04 ml at 0.6 mg/ml of pentobarbital molecule). Once reflexes had stopped, birds were intracardially perfused with PBS to remove blood, immediately followed by 4% paraformaldehyde PBS (PFA) to fix the brain. After perfusion, the brain was immediately extracted from the skull and postfixed during 24 h in 15-ml PFA.

The syrinx was extracted and weighed. For the winter group, the presence of the implant was confirmed and the T-filled implants were checked for the presence of remaining hormone inside. On the following day, brains were transferred to 15 ml of 30% sucrose solution. Once brains had sunk to the bottom of the vial, they were frozen on dry ice and stored at −80°C until used. When all brains had been collected, they were cut coronally on a Leica CM 3050S cryostat into four series of 30-μm-thick sections that were each distributed into four wells and these sections were stored in anti-freeze solution at −20°C.

Half a series (two non-adjacent wells; 240 μm between sections) were double-labeled in a single assay for PV and chondroitin sulfate, one of the main components of the PNNs, following a previously described protocol (Balmer et al., 2009; Cornez et al., 2015, 2017b, 2018). Briefly, sections were blocked in 5% normal goat serum (NGS) diluted in Tris-buffered saline (TBS) with 0.1% Triton X-100 (TBST) for 30 min. They were incubated overnight at 4°C in a mixture of 2 primary antibodies diluted in TBST: a mouse monoclonal anti-chondroitin sulfate antibody (CS-56, 1:500; C8035, Sigma-Aldrich) specific for the glycosaminoglycan portion of the chondroitin sulfate proteoglycans that are the main components of the PNN and a polyclonal rabbit anti-PV antibody (1:1000; ab11427, Abcam; RRID: AB_298032). Sections were then incubated at room temperature in a mixture of secondary antibodies diluted in TBST. A goat anti-mouse IgG coupled with Alexa Fluor 488 (green, 1:100, Invitrogen) was used to visualize PNN staining and a goat anti-rabbit IgG coupled with Alexa Fluor 546 (red, 1:200, Invitrogen) was used to visualize PV cells. Finally, sections were mounted on slides using TBS with gelatin and coverslipped with Vectashield containing DAPI (H-1500, Vector laboratories) that was used to confirm that PNN that were not surrounding PV-positive cells were localized around a cell nucleus.

### Nucleus volume quantification

Dense patterns of PV and chondroitin sulfate staining were used to quantify the volume of HVC, RA, and area X (Cornez et al., 2015). The borders of lMAN are however not clearly outlined by this staining and the volume of this nucleus could therefore not be determined. Photomicrographs of all stained sections containing the nuclei HVC, RA, or area X were acquired at 5× magnification and the volume of these nuclei was quantified as previously described (Cornez et al., 2017b). First, the area of the regions of interest (ROIs; in mm^2^) within each section was measured using the ImageJ software (NIH; https://imagej.nih.gov/ij). The volume of each ROI was estimated by multiplying the measured surface in each section by the distance between sections (240 μm) and then summing the results for all the sections. Finally, the mean volumes in the left and right hemispheres were calculated and these are the values reported in this article.

### PNN and PV quantification

The numbers of PV-positive cells (PV), cells surrounded by PNN (PNN) and PV-positive cells surrounded by PNN (PV+PNN) were counted in the four song control nuclei HVC, RA, area X, and lMAN. The boundaries of the ROIs were determined based on the bright PV and/or PNN staining except for lMAN where the precise boundaries of the nucleus could not be identified. Two photomicrographs were acquired on each brain side in two sections equally spaced in the rostro-caudal axis for each ROI. These photomicrographs were obtained with a Leica fluorescence microscope with a 40× objective and fixed settings. Each photomicrograph was entirely contained within the ROIs so that quantifying the entire image always sampled a similar area. The numbers of PV, PNN, and PV+PNN were consequently counted in the entire photomicrographs with the Image J software as previously described (Cornez et al., 2017b).

Briefly, for each ROI, a mean value was calculated for the left and right side of each section, which was subsequently averaged across sections to obtain the number of stained structures per counted surface in a given ROI. These numbers were converted in densities/mm^2^ and also used to compute the % PV surrounded by PNN (%PVwithPNN) and the % PNN surrounding PV (%PNNwithPV). Finally, the volume of each nucleus of each bird was used to estimate the total number of counted objects in the entire nucleus (except for lMAN) using the following formula: (number of counted object) × (nuclei volume/(counted area × section thickness)). This allowed us to obtain the total number of PV, PNN, and PV+PNN per nucleus.

### Statistics

As most data did not meet normality and/or homoscedasticity criteria, all statistical analyses were performed using non-parametric tests. Physiologic measurements obtained at brain collection (plasma T, mean testis weight, and syrinx weight), song measurements and brain measurements were analyzed using a Kruskal–Wallis one-way ANOVA to study the effect of age (seasons) across the five groups, without including the T-implanted winter group. Multiple comparisons by the mean rank test were used in *post hoc* analyses when the Kruskal–Wallis ANOVA was significant. Additionally, we analyzed the effect of T added during the winter on the same measurements using Mann–Whitney *U* tests, comparing T-treated and control winter birds. Variation in T concentrations over time were analyzed by a Friedman repeated measures ANOVA (days −5, +10, +24 compared with implant day) separately for the T-treated and the control winter birds. Differences between T-treated and control winter birds were explored at each time point using Mann–Whitney *U* tests. Significance level was set at *p *<* *0.05. All data are reported as mean ± SEM. Effect sizes were calculated using η^2^ for the Mann–Whitney tests and H η^2^ for Kruskal–Wallis ANOVA (as described in Tomczak and Tomczak, 2014).

## Results

### Physiologic and morphologic changes across development in canaries

**Figure 2.**
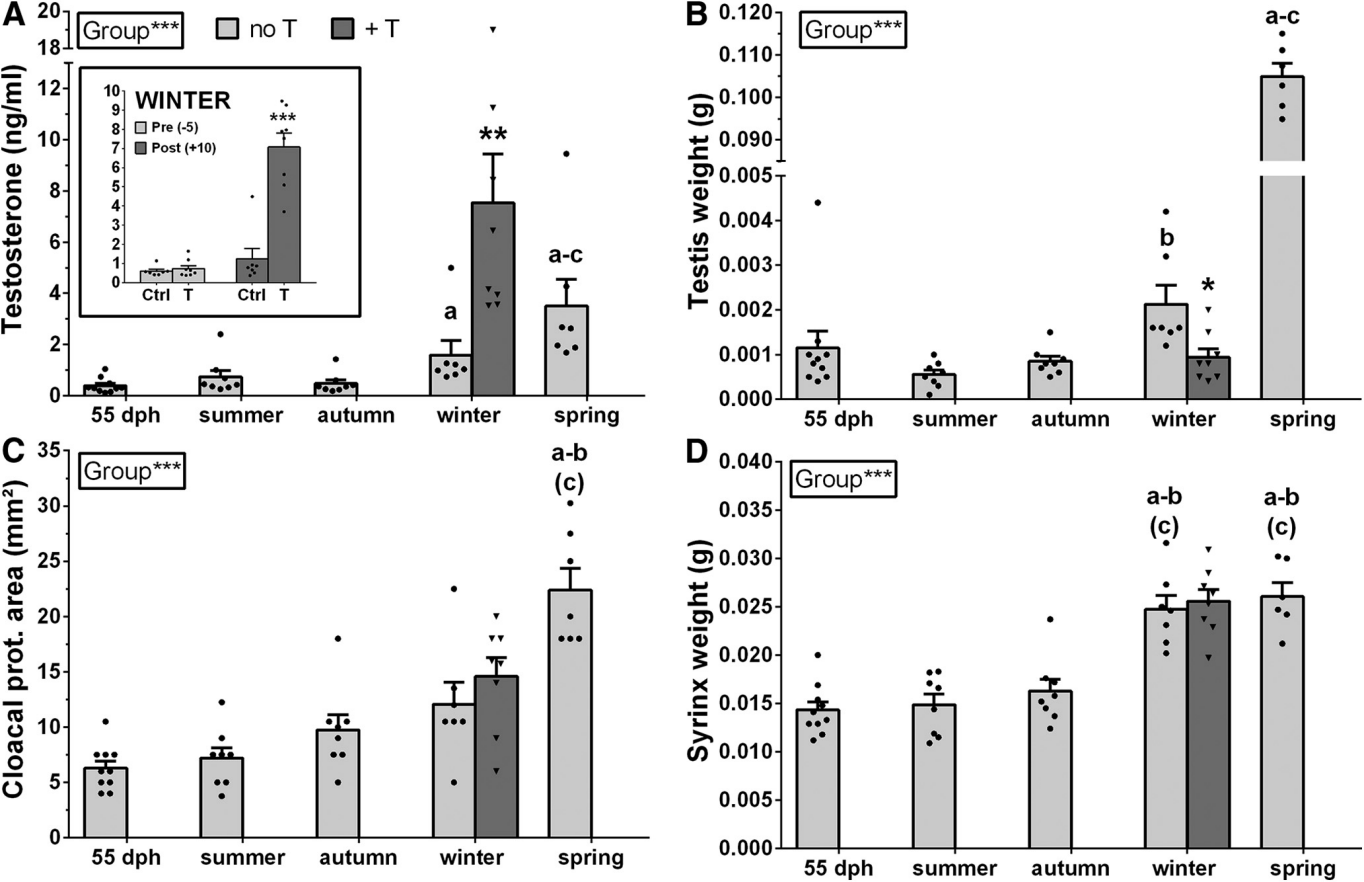
Plasma T concentrations (***A***), testis weight (***B***), cloacal protuberance area (***C***), and syrinx weight (***D***) during the first year of life of male canaries (light gray) and following T-treatment during the winter (dark gray). Significant differences between groups (seasons) as demonstrated by Kruskal–Wallis one-way ANOVAs are indicated in the inserts. Letters above bars indicate significant differences with the 55-dph group (a), the summer group (b), or the autumn group (c) as demonstrated by *post hoc* analyses. Letters in parentheses indicate a statistical trend (0.05 < *p *<* *0.10). Significant effects of T in the winter revealed by Mann–Whitney *U* test are indicated by asterisks. Additionally, the insert in panel ***A*** summarizes T plasma concentrations in control and T-treated birds during winter before and after implantation of the SILASTIC capsules. Measures of syrinx and mean testis weight were lost for one bird in the spring group, reducing sample size to *n* = 6; *p <* *0.05, **p <* *0.01, **p <* *0.001.

As observed in the Belgian Waterschlager Canary strain studied by Weichel et al. (1986), plasma T concentrations increased during development (*H*_(4,40)_ = 23.69, *p *<* *0.001, η^2^*_H_* = 0.51; Fig. 2*A*). This increase became most prominent toward the onset of the reproductive season. During the winter, T concentrations were already higher than at 55 dph (z = 2.85, *p *<* *0.05), as the birds broke the state of juvenile photorefractoriness, but as expected they reached their highest level in the spring after photostimulation, when they were significantly higher than at all other time points except winter (vs 55 dph: z = 4.19, *p *<* *0.001; vs summer: z = 2.82, *p *<* *0.05; vs autumn: z = 3.65, *p *<* *0.01). There was in parallel a similar increase of the mean testis weight (*H*_(4,39)_ = 26.40, *p *<* *0.001, η^2^*_H_* = 0.60; Fig. 2*B*) taking place at the same developmental period (winter vs summer: z = 3.29, *p *<* *0.05; spring vs 55 dph: z = 3.42, *p *<* *0.01; spring vs summer: z = 4.42, *p *<* *0.001; spring vs autumn: z = 3.45, *p *<* *0.01). The cloacal protuberance area, an indirect measure of androgen activity, also increased with age (*H*_(4,39)_ = 23.44, *p *<* *0.001, η^2^*_H_* = 0.51; Fig. 2*C*), but this change only became statistically significant in the spring (vs 55 dph: z = 4.36, *p *<* *0.001; vs summer: z = 3.69, *p *<* *0.01; vs autumn: z = 2.59, *p *<* *0.10). We also observed a significant increase of the syrinx weight (*H*_(4,39)_ = 25.18, *p *<* *0.001, η^2^*_H_* = 0.56; Fig. 2*D*) that was already significant during the winter (vs 55 dph: z = 3.74, *p *<* *0.01; vs summer: z = 3.29, *p *<* *0.05; vs autumn: z = 2.72, *p *<* *0.10) and was maintained in the spring (vs 55 dph: z = 3.65, *p *<* *0.01; vs summer: z = 3.18, *p *<* *0.05; vs autumn: z = 2.58, *p *<* *0.10).

T treatment during the winter significantly increased the blood T concentrations (*U* = 4, *N* = 15, *p *<* *0.01, η^2^ = 0.49) to a value that was even higher than in the next spring. T treatment reduced the mean testis weight (*U* = 6.5, *N* = 15, *p *<* *0.05, η^2^ = 0.40) presumably via a negative feedback blocking gonadotropin secretion. However, T treatment had no effect on the cloacal protuberance area (*U* = 18, *N* = 15, *p *>* *0.10, η^2^ = 0.08) or the syrinx weight (*U* = 24, *N* = 15, *p *>* *0.10, η^2^ = 0.01). This could relate to the fact that these structures had already reached a very large size (see above).

### Song development across seasons

Song analyses only included birds recorded from the summer (group 2) onwards since 55-dph males did not sing. One bird in the winter control subgroup did not sing and was consequently not included any of the song analyses, nor in all analyses of brain structures. As expected, singing behavior changed extensively over time. This concerned most of the song characteristics analyzed in this study confirming that song development involves modifications of multiple aspects of singing behavior including the motivation to sing, but also song quality and stereotypy.

**Figure 3.**
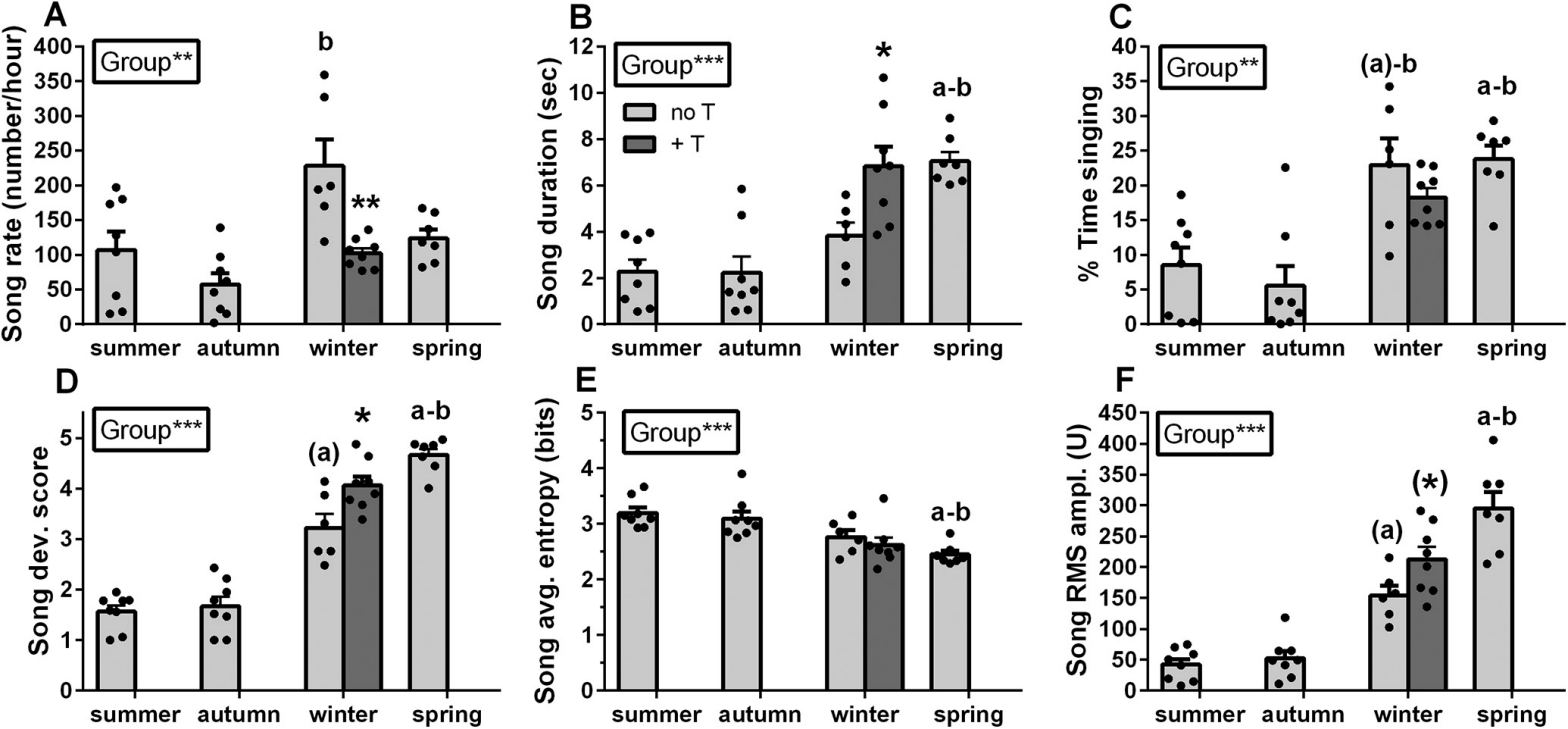
Changes in song rate (***A***), song duration (***B***), percentage of time spent singing (***C***), song developmental score (***D***), song entropy (***E***), and song RMS amplitude (***F***) of male canaries during the first year of life (light gray) and following T-treatment during the winter (dark gray). Significant differences between groups (seasons) as demonstrated by Kruskal–Wallis one-way ANOVAs are indicated in the inserts. Letters above bars indicate significant differences with the summer (a) and the autumn (b) group as demonstrated by *post hoc* analyses. Letters in parentheses indicate a statistical trend (0.05 < *p *<* *0.10). Significant effects of T in the winter revealed by Mann–Whitney *U* tests are shown by asterisks in the graph; *p <* *0.05, **p <* *0.01, **p <* *0.001.

Specifically, the song rate (number of songs/hour) varied significantly across seasons (*H*_(3,29)_ = 13.04, *p *<* *0.01, η^2^*_H_* = 0.32; Fig. 3*A*) with a significant increase observed in the winter compared with the autumn period (z = 3.60, *p *<* *0.01). There was also a progressive increase of the song duration (*H*_(3,29)_ = 17.71, *p *<* *0.001, η^2^*_H_* = 0.51; Fig. 3*B*) that became significantly longer at the onset of the breeding season compared with the previous summer and autumn (spring vs summer: z = 3.66, *p *<* *0.01; spring vs autumn: z = 3.69, *p *<* *0.01). Interestingly, the percentage of time spent singing was equally increased during both winter and spring compared with previous time points (*H*_(3,29)_ = 15.77, *p *<* *0.01, η^2^*_H_* = 0.43; spring vs summer: z = 2.73, *p *<* *0.05; spring vs autumn: z = 3.13, *p *<* *0.05; winter vs summer: z = 2.45, *p *<* *0.10; winter vs autumn: z = 2.83, *p *<* *0.05; Fig. 3*C*). T treatment during the winter decreased song rate (*U* = 2, *N* = 14, *p *<* *0.01, η^2^ = 0.55; Fig. 3*A*), but increased song duration (*U* = 7, *N* = 14, *p *<* *0.05, η^2^ = 0.32; Fig. 3*B*), without affecting the percentage of time singing (*U* = 16, *N* = 14, *p *>* *0.05, η^2^ = 0.07; Fig. 3*C*), so that singing behavior in this group became very similar to what was observed in the spring group.

We also quantified the development of song on a qualitative scale evaluating its progression toward crystallization. The song developmental score increased over time (*H*_(3,29)_ = 22.56, *p *<* *0.001, η^2^*_H_* = 0.43; Fig. 3*D*) and song crystallization started during the winter when the song developmental score (see Fig. 4 for representative spectrograms corresponding to each developmental score) already tended to be higher than during the summer (z = 2.51, *p *<* *0.10). Full crystallization was however only reached in the spring when the average score became significantly higher than during both the previous summer and autumn periods (vs summer: z = 4.02, *p *<* *0.001; vs autumn: z = 3.85, *p *<* *0.001). As previously described in canaries and other songbird species (Korsia and Bottjer, 1991; Alliende et al., 2010; Templeton et al., 2012), T accelerated song development during the winter so that developmental scores were higher in the T-treated than in the control birds (*U* = 8, *N* = 14, *p *<* *0.05, η^2^ = 0.29).

The song average entropy decreased with time (*H*_(3,29)_ = 16.76, *p *<* *0.001, η^2^*_H_* = 0.47; Fig. 3*E*) and was significantly lower during spring compared with the preceding summer and autumn periods (vs summer: z = 3.85, *p *<* *0.001; vs autumn: z = 3.00, *p *<* *0.05). However, T did not decrease song entropy during winter (*U* = 17, *N* = 14, *p *>* *0.10, η^2^ = 0.05). Additionally, various aspects of song quality changed over time as reflected by the change in song RMS amplitude (*H*_(3,29)_ = 22.27, *p *<* *0.001, η^2^*_H_* = 0.69; Fig. 3*F*), a measure of song loudness, that already tended to be higher in the winter compared with summer (z = 2.51, *p *<* *0.10) and was significantly higher in the spring compared with summer and autumn (vs summer: z = 4.05, *p *<* *0.001; vs autumn: z = 3.80, *p *<* *0.001). T also tended to increase this measure in the winter birds (*U* = 9, *N* = 14, *p *<* *0.10, η^2^ = 0.25). Similar results were found for three additional measures of the song loudness: the average power, the maximum power and the maximum amplitude. They increased over time, tended to be higher during the winter than during the previous summer and autumn (for the power measurements only), and were significantly higher at the onset of the breeding season in spring than during earlier periods (for details, see Table 2). However, these measures did not change following T treatment during the winter.

**Table 2:** Quantitative analyses of various aspects of the songs produced at different seasons by first year male canaries

Singing behavior	Summer	Autumn	Winter (no T)	Winter (+ T)	Spring	Kruskal–Wallis*H* (DF) (η²*_H_*)	Mann–Whitney*U* (η²)
First quartile frequency (Hz)	3160 ± 175	3364 ± 124	3768 ± 141	3933 ± 62	4034 ± 85^a-b^	*H*_(3)_ = 13.87** (0.35)	*U* = 13 (0.13)
third quartile frequency (Hz)	4293 ± 145	4340 ± 216	4846 ± 121	4857 ± 205	5019 ± 100 ^a-b^	*H*_(3)_ = 13.88** (0.36)	*U* = 17 (0.05)
5% frequency (Hz)	2144 ± 290	2188 ± 292	2985 ± 104	3100 ± 77	3277 ± 108 ^a-b^	*H*_(3)_ = 18.03*** (0.52)	*U* = 16 (0.07)
95% frequency (Hz)	5712 ± 273	5516 ± 145	5708 ± 195	5559 ± 60	5842 ± 131	*H*_(3)_ = 2.04 (0.11)	*U* = 21 (0.01)
Center frequency (Hz)	3742 ± 129	3899 ± 165	4267 ± 144	4385 ± 70	4513 ± 87 ^a-b^	*H*_(3)_ = 13.76** (0.35)	*U* = 16 (0.07)
Maximum frequency (Hz)	3911 ± 141	4039 ± 189	4416 ± 129	4536 ± 81	4443 ± 103	*H*_(3)_ = 8.04* (0.12)	*U* = 15 (0.09)
IQR bandwidth (Hz)	1132 ± 160	977 ± 128	1078 ± 53	924 ± 32	985 ± 46	*H*_(3)_ = 1.27 (0.15)	*U* = 8* (0.29)
90% bandwidth (Hz)	3568 ± 455	3328 ± 360	2723 ± 146	2459 ± 70	2566 ± 116	*H*_(3)_ = 6.80^(^*^)^ (0.07)	*U* = 13 (0.13)
Average power (dB)	32.4 ± 2.6	33.6 ± 2.1	44.9 ± 0.9 ^(a-b)^	47.0 ± 0.9	50.2 ± 0.9 ^a-b^	*H*_(3)_ = 22.03*** (0.68)	*U* = 15 (0.09)
Maximum power (dB)	64.0 ± 2.6	65.6 ± 2.4	77.8 ± 1.1 ^(a-b)^	79.6 ± 1.0	81.6 ± 1.2 ^a-b^	*H*_(3)_ = 21.45*** (0.66)	*U* = 15 (0.09)
Maximum amplitude (U)	373 ± 82	511 ± 137	1511 ± 177 ^(a)^	1923 ± 208	2348 ± 221 ^a-b^	*H*_(3)_ = 21.73*** (0.67)	*U* = 14 (0.11)

The table shows the mean ± SEM of various measures of power distribution across frequencies (5%, first quartile, center, third quartile, 95%), of frequency at which the maximum power occurred (max frequency), of the bandwidth of this distribution (IQR and 90% range), and of three additional measures of vocalization loudness (average power, maximum power, and maximum amplitude). The last two columns present the statistical results of the Kruskal–Wallis ANOVA for the seasonal effect and the results of the Mann–Whitney tests of the effect of T during the winter. Results of significant *post hoc* tests are labeled by the letters a and b indicating a significant different by comparison with the summer and autumn respectively. Effect size is indicated in parentheses for each test. Levels of significance are indicated as follows: (*)*p *<* *0.10, *p <* *0.05, **p <* *0.01, ***p <* *0.001.

Additionally, the power distribution across frequencies in the songs changed over time. There was an overall increase of the 5%, first quartile, center and third quartile frequencies without changes of the 95% frequency showing a displacement of the vocalization power toward the higher frequencies, or in other words an increased percentage of the power was only expressed above the corresponding frequencies. The *post hoc* analyses of all these measures showed a significant increase in the spring compared with the summer and autumn, but not yet in the winter. None of these measures was affected by T treatment in the winter. This pattern of power displacement probably leads to a narrowing of the song bandwidth that seems to be confirmed by the analysis of the 90% bandwidth (the distribution of 90% of the power) that tended to decrease over time. In contrast, the IQR range bandwidth (50% of the power distribution between the first and third quartile frequency) did not change over time, while both the first and third quartile frequencies increased. However, the IQR bandwidth was significantly decreased by T. Finally, the maximum frequency, which is the frequency at which the maximum power occurs also increased with time, but was not affected by the T treatment during the winter (for a detail of results, see Table 2).

### The volume of song control nuclei increases during the winter before sexual maturity

**Figure 5.**
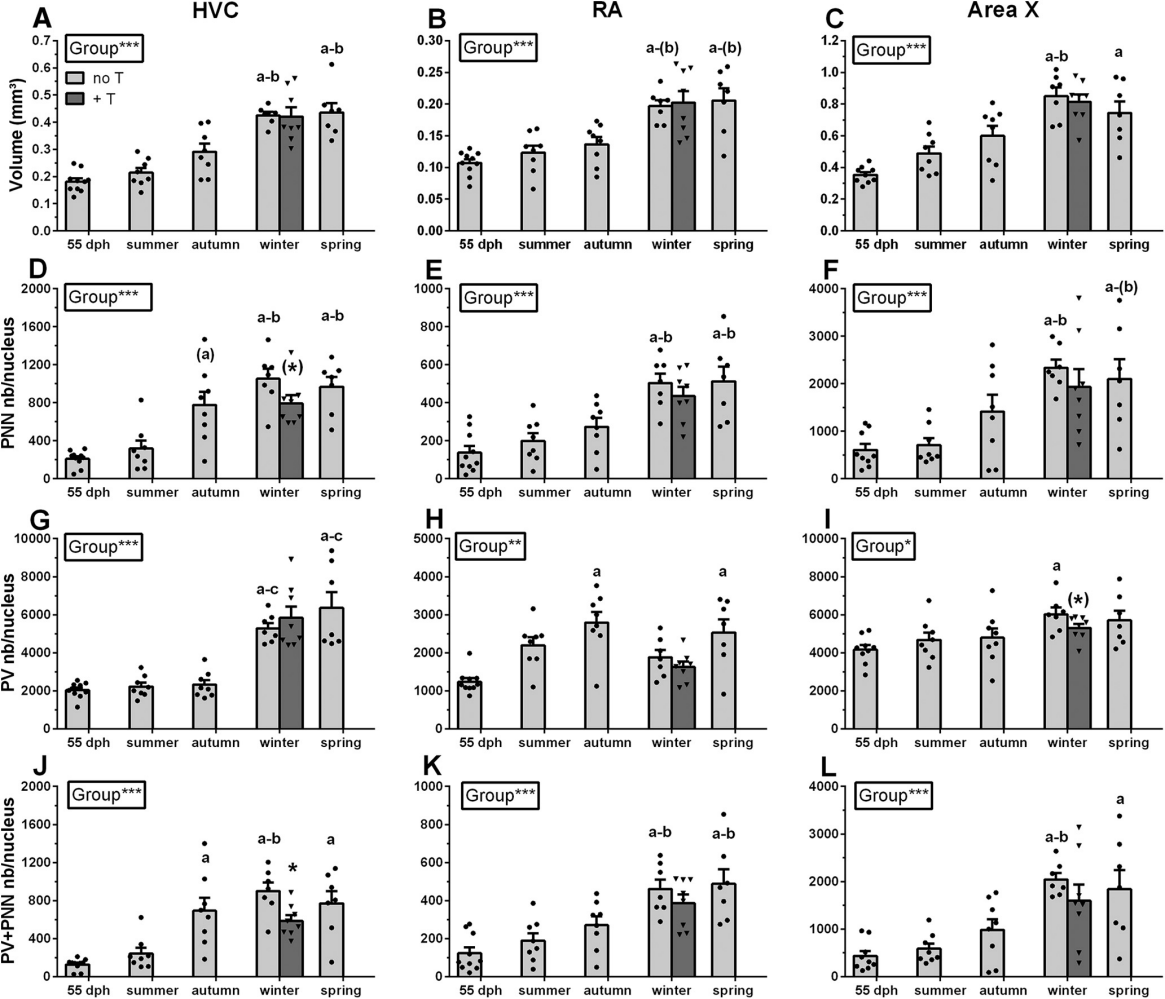
Changes of the volume of song control nuclei (***A–C***), the number of PNNs per nucleus (***D–F***), the number of PV-immunoreactive neurons per nucleus (***G–I***), and the number of PV-PNNs per nucleus (***J–L***) in male canaries during ontogeny (light gray) and following T-treatment during winter (dark gray). The very faint PNN and PV staining in area X of one 55-dph bird did not allow the delineation of this nucleus and determination of its volume, so that the total numbers of PNN and PV in this nucleus could not be computed. The final sample size for these measures in this group is thus reduced to *n* = 9. Significant differences between groups (seasons) as demonstrated by Kruskal–Wallis one-way ANOVAs are indicated in the inserts. Letters above bars indicate significant differences with the 55-dph group (a), the summer group (b) and the autumn group (c), as demonstrated by the *post hoc* analyses. Letters in parentheses indicate a trend (0.05 < *p *<* *0.10). Significant effects of T treatment in winter revealed by Mann Whitney *U* tests are shown in the graph; (*)*p *<* *0.10*, *p *<* *0.05, **p <* *0.01, **p <* *0.001.

The song control nuclei volume increased over time (HVC: *H*_(4,40)_ = 29.22, *p *<* *0.001, η^2^*_H_* = 0.66; RA: *H*_(4,40)_ = 22.86, *p *<* *0.001, η^2^*_H_* = 0.48; area X: *H*_(4,40)_ = 24.19, *p *<* *0.01, η^2^*_H_* = 0.52; Fig. 5*A–C*). The increase in volume of HVC, RA, and area X followed a fairly similar time course: during the winter, these volumes were already significantly larger than at 55 dph (HVC: z = 4.24, *p *<* *0.001; RA: z = 3.85, *p *<* *0.01; area X: z = 4.36, *p *<* *0.001) and during the summer, except for RA (HVC: z = 3.28, *p *<* *0.05; RA: z = 2.79, *p *<* *0.10; area X: z = 2.87, *p *<* *0.05). The maximum volume of all three nuclei was in fact already attained in the winter and it stayed at a similar level in the following spring, thus maintaining the significant differences with volumes measured at 55 dph (HVC: z = 4.19, *p *<* *0.001; RA: z = 3.70, *p *<* *0.01; area X: z = 3.59, *p *<* *0.01) and to some extent in summer (HVC: z = 3.24, *p *<* *0.05; but RA: z = 2.65, *p *<* *0.10, and area X: z = 2.11, *p *>* *0.10). T treatment during winter did not increase the volume of the song control nuclei, further suggesting they had already reached their maximal value (ceiling effect).

### PNN develops around PV-interneurons between summer and winter in HVC

**Figure 6.**
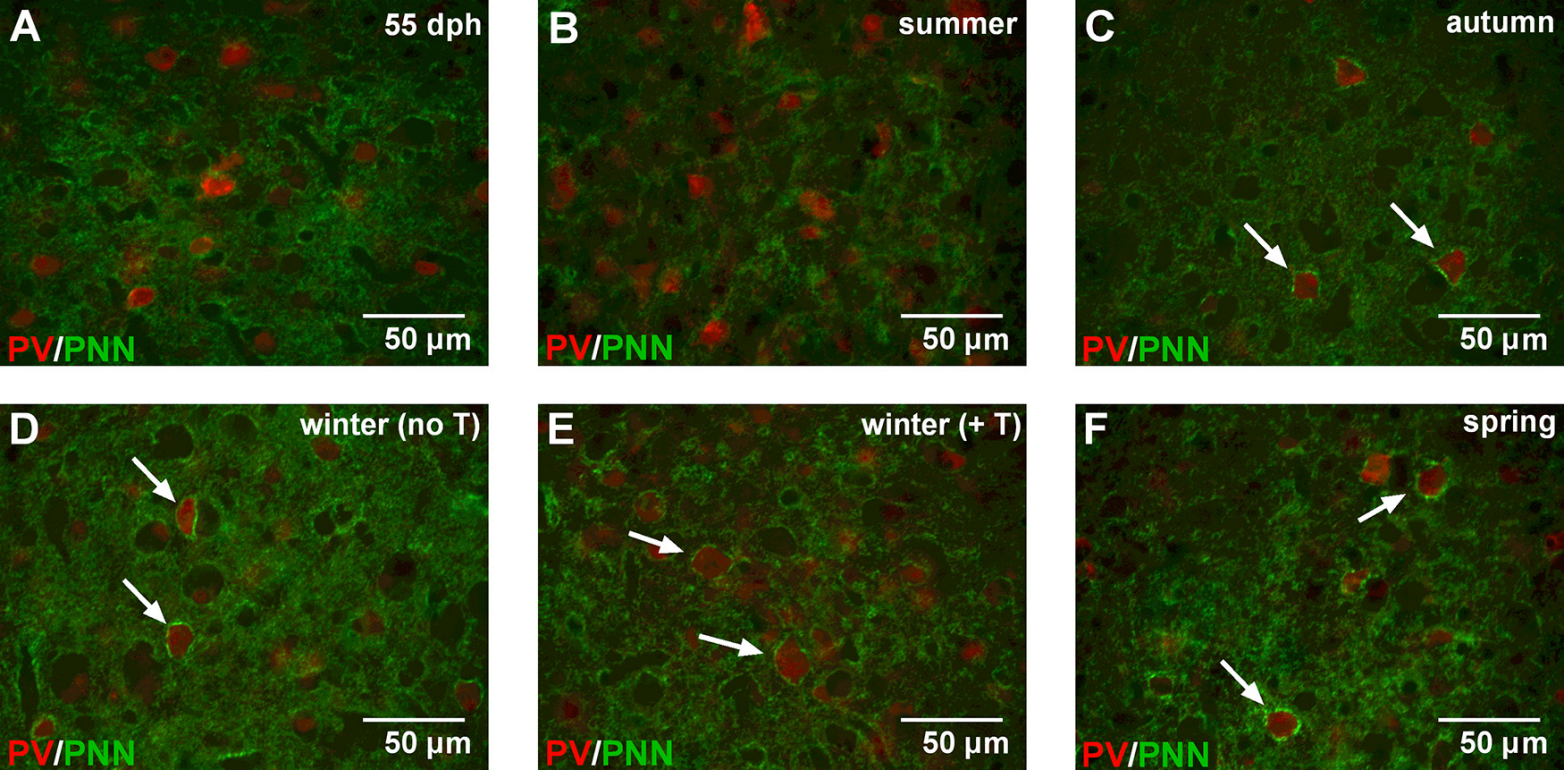
***A–F***, Representative photomicrographs of the double-staining for PV (red) and PNN (green) in HVC of each experimental group. White arrows indicate PV-positive neurons surrounded by PNN.

In HVC, the number of PNNs progressively increased during development (*H*_(4,40)_ = 25.11, *p *<* *0.001, η^2^*_H_* = 0.55; Fig. 5*D*) and tended already in autumn to differ from the 55-dph period (z = 2.70, *p *<* *0.10) (see Fig. 6 for representative photomicrographs). The number of PNN then continued to increase being significantly higher in winter than at 55 dph and in the summer (vs 55 dph: z = 3.89, *p *<* *0.001; vs summer: z = 3.24, *p *<* *0.05). The total number of PNNs per HVC then remained stable until the spring, being significantly different from values at 55 dph and in summer (vs 55 dph: z = 3.58, *p *<* *0.01; vs summer: z = 2.93, *p *<* *0.05).

The number of PV-interneurons in HVC also increased over time (*H*_(4,40)_ = 26.82, *p *<* *0.001, η^2^*_H_* = 0.59; Fig. 5*G*), but this increase was more abrupt and had not yet been initiated in the autumn. The number of PV-interneurons was significantly higher in the winter and spring compared with all previous periods including the autumn (vs 55 dph: z = 3.55, *p *<* *0.01; vs summer: z = 3.10, *p *<* *0.05; vs autumn: z = 3.08, *p *<* *0.05; spring vs 55 dph: z = 3.77, *p *<* *0.01; vs summer: z = 3.31, *p *<* *0.01; vs autumn: z = 3.29, *p *<* *0.05).

The number of PNN surrounding PV-interneurons (PV+PNN) similarly increased over time (*H*_(4,40)_ = 25.83, *p *<* *0.001, η^2^*_H_* = 0.57; Fig. 5*J*) and was significantly higher than at 55 dph from autumn until spring (vs autumn: z = 3.27, *p *<* *0.05; vs winter: z = 4.12, *p *<* *0.001; vs spring: z = 3.57, *p *<* *0.01). PV+PNN was also higher than in the summer during the winter (z = 2.99, *p *<* *0.05). Surprisingly, the number of PV+PNN in HVC was significantly reduced by the T-treatment in winter (*U* = 7, *N* = 15, *p *<* *0.05, η^2^ = 0.38) and there was a similar trend for the number of PNN (*U* = 13, *N* = 15, *p *<* *0.10, η^2^ = 0.19).

We found similar results when analyzing the density (number per mm^2^) of PNN, PV+PNN and PV in HVC (Table 3): they all increased over time. PNN and PV+PNN were significantly higher in autumn and winter compared with the 55-dph period, but this difference disappeared in the spring when the PNN density only tended to be higher (*p* < 0.10) than during the 55-dph period. As observed for the total number of PV, the PV density was significantly higher in the winter and spring, but compared with autumn only.

**Table 3:** Analysis of the densities (numbers/mm^2^) of PNN, PV and PV+PNN, % PV surrounded by PNN and % PNN located around PV in the three SCS nuclei

Brain	55 dph	Summer	Autumn	Winter (no T)	Winter (+ T)	Spring	Kruskal–Wallis*H* (DF) (η²*_H_*)	Mann–Whitney*U* (η²)
HVC								
PNN density (/mm²)	34.8 ± 4.7	42.8 ± 9.4	76.9 ± 10.6^a^	73.8 ± 6.8^a^	58.8 ± 7.7	67.2 ± 6.9^(a)^	*H*_(4)_ = 17.28** (0.32)	*U* = 14 (0.16)
PV density (/mm²)	347 ± 28	311 ± 8	247 ± 23	374 ± 21^c^	414 ± 49	440 ± 50^c^	*H*_(4)_ = 16.74** (0.31)	*U* = 13 (0.19)
PV+PNN density (/mm²)	20.9 ± 3.4	32.6 ± 6.3	68.9 ± 10.6^a^	63.0 ± 5.4^a^	43.5 ± 5.4	53.1 ± 9.1	*H*_(4)_ = 21.53*** (0.44)	*U* = 10* (0.28)
% PV with PNN	6.6 ± 1.0	10.8 ± 2.4	32.2 ± 8.2^a-b^	17.4 ± 2.0^a^	10.8 ± 1.5	13.8 ± 2.9	*H*_(4)_ = 20.04*** (0.40)	*U* = 6* (0.41)
% PNN with PV	58.3 ± 4.3	81.0 ± 4.4	89.3 ± 2.5^a^	85.7 ± 1.8^a^	75.2 ± 5.2	78.1 ± 10.6^(a)^	*H*_(4)_ = 18.94*** (0.37)	*U* = 13.5 (0.18)
RA								
PNN density (/mm²)	37.1 ± 7.8	45.78 ± 7.7	58.8 ± 9.2	76.3 ± 6.5^a^	66.0 ± 7.3	73.8 ± 6.6^a^	*H*_(4)_ = 13.85** (0.22)	*U* = 18 (0.08)
PV density (/mm²)	355 ± 30	545 ± 50^(a)^	626 ± 70^a^	288 ± 29^b-c^	244 ± 12	361 ± 31^(c)^	*H*_(4)_ = 22.59*** (0.47)	*U* = 18 (0.08)
PV+PNN density (/mm²)	33.1 ± 7.2	43.6 ± 7.8	58.1 ± 8.9	69.7 ± 6.2^(a)^	57.3 ± 5.3	70.5 ± 6.6^a^	*H*_(4)_ = 13.53** (0.22)	*U* = 15 (0.14)
% PV with PNN	10.6 ± 2.7	8.5 ± 1.6	10.7 ± 2.2	25.4 ± 2.9^a-b^	24.0 ± 2.5	21.0 ± 3.2	*H*_(4)_ = 17.27** (0.32)	*U* = 26.5 (0.00)
% PNN with PV	90.1 ± 3.9	94.6 ± 2.8	99.2 ± 0.8	91.5 ± 2.9	88.8 ± 4.1	95.8 ± 3.1	*H*_(4)_ = 5.85 (0.00)	*U* = 24 (0.01)
Area X								
PNN density (/mm²)	48.8 ± 8.0	42.1 ± 5.6	63.1 ± 12.5	82.9 ± 5.0^(b)^	71.1 ± 12.6	83.8 ± 13.4	*H*_(4)_ = 11.88* (0.17)	*U* = 20 (0.05)
PV density (/mm²)	355 ± 14	290 ± 8	244 ± 14^a^	216 ± 14^a-(b)^	198 ± 9	235 ± 15^a^	*H*_(4)_ = 25.49*** (0.56)	*U* = 22.5 (0.02)
PV+PNN density (/mm²)	35.4 ± 6.8	34.8 ± 4.1	44.3 ± 8.8	73.0 ± 4.4^a-b^	58.8 ± 12.2	73.0 ± 13.7	*H*_(4)_ = 15.44** (0.27)	*U* = 21 (0.04)
% PV with PNN	9.8 ± 1.7	12.1 ± 1.4	19.5 ± 4.5	34.4 ± 2.4^a-b^	30.2 ± 6.1	31.4 ± 5.8^a^	*H*_(4)_ = 20.73*** (0.42)	*U* = 24 (0.01)
% PNN with PV	72.5 ± 6.1	84.7 ± 4.1	68.9 ± 4.5	88.6 ± 3.8^(c)^	77.5 ± 7.5	83.4 ± 5.0	*H*_(4)_ = 10.39* (0.13)	*U* = 19.5 (0.06)
LMAN								
PNN density (/mm²)	38.9 ± 7.7	39.9 ± 10.1	53.0 ± 10.2	61.4 ± 11.5	55.9 ± 8.1	63.9 ± 15.5	*H*_(4)_ = 3.97 (–0.06)	*U* = 25.5 (0.00)
PV density (/mm²)	338 ± 23	418 ± 29	490 ± 49	620 ± 42^a^	718 ± 46	649 ± 37^a^	*H*_(4)_ = 23.86*** (0.51)	*U* = 17.5 (0.09)
PV+PNN density (/mm²)	19.2 ± 8.4	34.8 ± 9.1	47.9 ± 8.9	54.7 ± 9.8	50.8 ± 7.1	60.5 ± 15.4^(a)^	*H*_(4)_ = 10.76* (0.14)	*U* = 26.5 (0.00)
% PV with PNN	6.6 ± 3.5	9.1 ± 2.7	11.0 ± 2.7	8.74 ± 1.4	7.3 ± 1.2	9.7 ± 2.8	*H*_(4)_ = 5.33 (–0.02)	*U* = 22.5 (0.02)
% PNN with PV	41.0 ± 14.0	87.4 ± 4.6	92.8 ± 4.0	91.1 ± 3.6	91.6 ± 2.8	91.5 ± 7.0	*H*_(4)_ = 8.15 (0.06)	*U* = 26 (0.00)

The table shows the mean ± SEM of the different measures. The last two columns present the statistical results of the Kruskal–Wallis ANOVA for the seasonal effect and the results of the Mann–Whitney tests of the effect of T during the winter. Results of significant *post hoc* tests are labeled by the letters a, b, and c indicating a significant different by comparison with the 55 dph, summer, and autumn, respectively. Letters in parenthesis indicate a trend (0.05 < *p *<* *0.10). Effect size is indicated in parenthesis for each test. Levels of significance are indicated as follows: (*)*p *<* *0.10, *p <* *0.05, **p <* *0.01, ***p <* *0.001.

The increase in PV density and number occurred only during the winter, while the increase of PNN and PV+PNN densities and numbers started already in autumn. It is hence likely that the development of PNN occurs first around some pre-existing PV-expressing neurons in the autumn, to develop thereafter around additional neurons that begin to express PV in winter and spring. This conclusion is supported by the significant increase of the % PV surrounded by PNN observed in autumn (comparison to 55 dph and the summer period) and to some extent in winter (significant comparison with 55 dph only), which is no longer present during the spring. Note that T significantly decreased the density of PV+PNN as well as the %PV with PNN, which somehow mimics what takes place in the spring. Finally, there was also an increase of the % PNN surrounding PV over time, so that this percentage was significantly larger from autumn until spring when compared with 55 dph. Only 58% of PNN were surrounding PV at 55 dph, whereas in most cases, in older birds >80% PNN were located around PV-expressing. This suggests that PNN surround a considerable amount of different HVC cell types at earlier developmental stages (for detail of results, see Table 3).

### PNN develop around PV-interneurons during the winter in RA and area X

In RA and area X, there was a very similar timing of PNN development around PV-expressing neurons: the only differences between nuclei concerned the degree of significance between groups in *post hoc* analyses. Overall, PNN developed around PV-interneurons during the winter preceding the first breeding season. The number of PNN and of PV+PNN differed across time points in RA [PNN (Fig. 5*E*): *H*_(4,40)_ = 23.02, *p *<* *0.001, η^2^*_H_* = 0.49; PV+PNN (Fig. 5*K*): *H*_(4,40)_ = 23.84, *p *<* *0.001, η^2^*_H_* = 0.51] and in area X [PNN (Fig. 5*F*): *H*_(4,40)_ = 19.43, *p *<* *0.001, η^2^*_H_* = 0.38; PV+PNN (Fig. 5*L*): *H*_(4,40)_ = 21.09, *p *<* *0.001, η^2^*_H_* = 0.43]. *Post hoc* tests further indicated that PNN and PV+PNN numbers increased specifically during winter and were significantly higher than during the 55-dph period in RA (PNN: z = 3.83, *p *<* *0.01; PV+PNN: z = 3.86, *p *<* *0.01) and in area X (PNN: z = 3.55, *p *<* *0.01; PV+PNN: z = 3.87, *p *<* *0.01) as well as in the summer (in RA: PNN: z = 3.05, *p *<* *0.05; PV+PNN: z = 3.85, *p *<* *0.05; in area X: PNN: z = 3.14, *p *<* *0.05; PV+PNN: z = 3.13, *p *<* *0.05). Levels remained high in the spring as illustrated by similar significant differences with spring and the 55 dph (PNN: z = 3.58, *p *<* *0.01; PV+PNN: z = 3.86, *p *<* *0.01) and summer (PNN: z = 3.05, *p *<* *0.05; PV+PNN: z = 2.85, *p *<* *0.05) in RA, and in area X between the spring group and the 55-dph group (PNN: z = 3.00, *p *<* *0.05; PV+PNN: z = 3.15, *p *<* *0.05). There was however no significant difference between the spring and the summer in area X (PNN: z = 2.61, *p *<* *0.10; PV+PNN: z = 2.42, *p *>* *0.10).

The density of PNN and PV+PNN changed over time, and was significantly higher during winter and spring compared with the 55-dph period in RA (only a trend (*p *<* *0.10) for the PV+PNN density in the winter group). In area X, there was also a change in the density of PNN and of PV+PNN, but the *post hoc* analyses showed that the increase of the PV+PNN density occurred in the winter group, when numbers were higher than in the 55 dph and summer groups only. This difference was lost in the spring even if absolute values remained very similar. This is probably due to the higher variability in spring. PNN density in area X also changed over time, but *post hoc* analyses identified only a trend for a difference between the winter group and the summer group (for detail of results, see Table 3). Additionally, the % PV surrounded by PNN changed over time in both RA and area X, and was significantly higher in the winter group compared with the 55 dph and summer group, as well as in the spring group compared with the 55-dph group in area X only. Again, for this measure, the mean %PVwithPNN of the winter and spring group was very similar in both RA and area X, but the variability was higher in the spring group (for detailed results, see Table 3). This increase that occurs in the winter at the same time as the increase in the number of PNN and of PV+PNN suggests that the addition of PNN is the consequence of their development around PV-interneurons.

Even if there was a very similar timing of PNN development around PV-interneurons in RA and area X, these song control nuclei displayed specific patterns of change in the numbers and densities of PV-expressing neurons. In both nuclei, the number and density of PV-interneurons changed significantly over time [RA-PV numbers (Fig. 5*H*): *H*_(4,40)_ = 18.13, *p *<* *0.01, η^2^*_H_* = 0.35; area X-PV numbers (Fig. 5*I*): *H*_(4,40)_ = 12.56, *p *<* *0.05, η^2^*_H_* = 0.19; for density results, see Table 3]. *Post hoc* analyses showed a peak in the number of PV-interneurons occurring in the autumn and spring in RA, that were significantly higher than in the 55-dph group (autumn: z = 3.93, *p *<* *0.001; spring: z = 3.07, *p *<* *0.05; for the corresponding results on densities, see Table 3). In area X, the pattern of developmental changes was more similar to what happened in HVC, but at a lower magnitude. The *post hoc* analyses only showed that the number of PV increased in the winter group as compared with the 55-dph group (for detailed results on densities, see Table 3).

Finally, there were no changes in the proportion of PNN that are located around PV-expressing neurons in RA. This measure exceeded 90% at all developmental stages (Table 3). In area X, this measure significantly changed over time, but no significant differences were revealed in the *post hoc* analyses (Table 3). Interestingly, T treatment during winter did not significantly affect the number, density or proportions of all these measures.

### PNN expression in lMAN does not change during ontogeny

Since lMAN volume could not be determined in our material, PNN and PV could only be quantified as densities (numbers per mm^2^). Contrary to what had been observed in the three other song control nuclei, no change with age could be detected in lMAN in the density of PNN (*H*_(4,40)_ = 3.97, *p *=* *0.411, η^2^*_H_* = 0.06), and there was only a minimal change in the density of PV+PNN (*H*_(4,40)_ = 10.76, *p *=* *0.29, η^2^*_H_* = 0.14; Table 3). The *post hoc* tests failed to detect any significant difference associated with this small overall change in PV+PNN density; there was only a statistical tendency for increase in the spring group compared with the 55-dph birds. However, like in other nuclei, the density of PV-positive neurons increased with age (*H*_(4,40)_ = 23.86, *p *<* *0.001, η^2^*_H_* = 0.51) reaching a peak during the winter. The percentage of PV neurons surrounded by PNN was small and did not change with age while in contrast nearly all PNN were located around PV neurons regardless of the age of the birds. The smaller average percentage observed at 55 dph relates to a subgroup of subjects but was not sufficient to induce any statistically validated difference. T addition during the winter did not affect any of these measures.

## Discussion

We explored in parallel song learning and the development of PNN in four song control nuclei, HVC, RA, area X, and lMAN, of juvenile canaries from fledging until their first breeding season. In zebra finches PNN start developing around the end of the sensory and the beginning of the sensorimotor stage of vocal learning (Cornez et al., 2018) but the rapid maturation in this species associated with the overlap between sensory and sensorimotor phases of learning (Williams, 2004) did not allow us to link precisely the increase in PNN to a specific aspect of song development. Moreover, singing behavior was not recorded in this study because on zebra finches were raised in a large aviary so that relationships between PNN development and song learning had to be based on previous studies of song development of this species (Brainard and Doupe, 2002; Williams, 2004). Although there is also some overlap between the sensory and sensorimotor phases of song learning in canaries (see Introduction), the present study provided a better opportunity to directly relate neurobiological processes in the song control system with specific song developmental stages because sensorimotor learning in canaries lasts longer and extends from ∼60 dph until one year of age. We demonstrate that the development of PNN mostly takes place during the sensorimotor phase of song learning so that it is probably involved in song crystallization. However, the emergence of PNN peaks during the winter presumably before song crystallization is completed. Finally, we confirm that T accelerates song crystallization during the winter, but without inducing any detectable increase of PNN numbers or density in the song control system, probably because PNN have already reached a plateau that is sufficient to support song crystallization.

### Song crystallizes during winter and early spring in juvenile canaries

We analyzed the development of song during ontogeny in juvenile male canaries of the Fancy Fife strain that had not been studied before. Song development was evaluated qualitatively with the use of a score capturing changes across all stages of the sensorimotor development. This approach, in combination with an automated analysis of song characteristics, identified a set of song characteristics that start changing during winter (e.g., percentage time spent singing, RMS amplitude) as birds break juvenile photorefractoriness and become photosensitive (Williams et al., 1987; Follett, 1991; Ball and Wade, 2013). These song features then continue to evolve to reach their full development at the onset of the first breeding season in spring. A second set of song parameters was found to change only at the onset of the breeding season when canaries experience long days and usually attain full breeding status. This was the case for the song entropy, the power distribution across frequencies that was displaced toward higher frequencies and the frequency at which the maximum power occurs.

It is interesting that the rate of singing increased during ontogeny before other features that characterize the mature song since it is well established that the birds need to practice their song during the sensorimotor period. Our previous work analyzing the endocrine control of singing in canaries via stereotaxic implantation of T or anti-androgens directly into the brain also demonstrated that the singing motivation is largely controlled at the level of the medial preoptic area whereas other features of song such as the variability of bandwidth or of entropy are controlled by T action in HVC or RA (Alward et al., 2013, 2016). We additionally showed that in males and females a systemic treatment with T increases the singing motivation within a few days while the adult song structure develops more progressively (Alward et al., 2013, 2016; Madison et al., 2015) and that correlatively, the morphologic effects of T become visible in the medial preoptic nucleus before they do so in HVC (Shevchouk et al., 2017, 2019). The sequence observed here at the behavioral level in developing young birds fits with the results of these previous studies.

The song rate largely increased during winter presumably as the birds became photosensitive making them able to respond to a variety of reproductively significant stimuli such as green vegetation or a sexual partner (Voigt et al., 2011). However, song rate decreased in spring to levels present at the onset of song learning. This can probably be explained by the fact that juvenile canaries sing more short songs during winter, and fewer, but longer, songs during spring compared with preceding periods of development. Together, these data indicate that song crystallization begins during winter to be fully completed at the onset of the spring breeding season, and this process involves changes of different song characteristics at specific periods. Interestingly, the syrinx already increased to maximal weight during winter when plasma T concentrations were only slightly increased, and still far lower than in spring. This suggests that this organ presumably becomes especially sensitive to androgens at early stages, and that the syrinx is fully developed before birds crystallize their song, a time sequence that might in fact be mandatory.

### Development of PNN correlates with sensorimotor song learning

In canaries, memorization of the tutor song is completed during the summer when the breeding season of the previous generation ends and adult males generally stop singing (Leitner et al., 2015). The earliest tendency toward an increase of PNN in HVC was detected during the autumn, and still was not statistically significant. The main, significant increase of PNN and of PV+PNN occurred during the winter. Sensory learning had been completed for at least several weeks or months at that time, in part because adult males that could serve as tutors had stopped singing, and brains of the autumn group were collected at the end of October. The start of PNN increase is thus presumably dissociated from the end of the sensory vocal learning stage. In zebra finches, PNN increase starts at 60 dph, which corresponds to the end of the sensory learning. It is thus difficult to definitively conclude in this species that the development of PNN is not involved in the closing of the sensitive period for sensory learning, even if PNN numbers continue to increase until adulthood (Balmer et al., 2009; Cornez et al., 2018). The present study rules this out and confirms that the PNN increase occurs during sensorimotor learning only and is possibly associated with the end of the song learning phase.

### Development of PNN slightly precedes full song crystallization

Because the number of PNN and of PV+PNN increased mostly during the winter and did not increase further in the spring, we suggest that their development occurs specifically during the transition from plastic to crystallized song. One could have thought that PNN development would only be completed after the full song crystallization to limit further changes in song control system connectivity as well as in song structure. However, PNN numbers and densities already reached their maximum during the winter when some song parameters, such as the developmental score and the RMS amplitude, had not yet reached their maximum. PNN full development thus preceded the development of a song typical of adult males and might be considered as a neural mechanism supporting early steps of song crystallization.

The percentage of time spent singing increased during the winter and remained at the same high level in the spring, a pattern similar to the change in PNN and PV+PNN numbers. It is however unlikely that PNN development in the song control system could be specifically tied to the control of this aspect of song that only depends on the song numbers and duration. It is more likely that PNN development during winter allows the progressive song crystallization by supporting stronger synaptic connections between PV-interneurons and putative projection neurons, which allows singing of accurately repeated syllables within phrases. Already during winter, some songs or part of songs contain phrases with accurately repeated syllables. The repetition accuracy and the precise utterance of syllables that were considered as two main criteria in establishing the developmental score are already partly present in songs obtaining a score of four during the winter.

Song crystallization induced by T in castrated adult males was associated with an increase of PNN in brains collected after 24 d while song was already crystallized after 10 d (Cornez et al., 2017a), suggesting that PNN development follows song crystallization. However, in females, T induced PNN development after a latency between 9 and 21 d (Cornez et al., 2017a). It is thus possible that in adult males also the T-induced PNN increase takes place at the beginning of song crystallization. Detailed time course studies of PNN development and song crystallization following exposure to T would be needed to more precisely determine the sequence of these events.

### T accelerates song development in winter without increasing PNN numbers

T increases singing rate and promotes the development of song features typically seen in the song of mature males (e.g., longer duration, higher energy) in castrated male and female canaries (Cornez et al., 2017a; Vellema et al., 2019). We also previously showed that T increases the number of PNN in HVC, RA, and area X in adult female and castrated male canaries (Cornez et al., 2017a). In contrast, T applied here in juvenile males during their first winter tended to decrease the number of PNN and significantly decreased the number of PV+PNN in HVC, while it increased song duration and the song developmental score. As the full development of PNN appears to precede the full development of the adult song, the lack of increase after T treatment might reflect a ceiling effect, but why would a decrease be observed remains an open question.

### Time course of PNN and PV development in HVC compared with other song control nuclei

A slight, non-significant increase in the number of PNN and of PV+PNN was already present in autumn in HVC, but not in RA nor area X. HVC has been shown to provide trophic signals to RA and area X that are responsible, at least in part, for the growth of these nuclei (Wissman and Brenowitz, 2009). PNN development similarly might occur with a delay between HVC and its two targets. How a trans-synaptic control of PNN formation would take place cannot yet be specified but PNN formation could be activity dependent (Hensch, 2004; Balmer et al., 2009) and therefore rely, at least in part, on inputs from HVC. It is alternatively possible that PNN formation is simply induced by the local action of T and that RA and area X just react more slowly to this endocrine stimulus. Stereotaxic implantation of T in or near all these nuclei should permit to distinguish between these possibilities.

It is finally remarkable that these changes were not observed in lMAN. This is at first sight somewhat surprising since lMAN has been demonstrated to play a key role in song learning, during ontogeny (Bottjer et al., 1984; Scharff and Nottebohm, 1991) and in adult seasonal species such as canaries when birds modify their song in the fall (Alliende et al., 2017), namely, by generating song variability that is essential for sensorimotor learning (Kao et al., 2005; Andalman and Fee, 2009). In contrast, PNN density was reported to change with age in zebra finches with the major increase occurring between 30 and 50 dph, that is before or around the end of the sensory period of song learning (Cornez et al., 2018). The precise end of sensory learning period has not been determined in canaries but should be somewhere between 50 and 100 dph (Leitner et al., 2015). Since the youngest canaries sampled here were 55 dph, it cannot be excluded that all samples were collected in the present study after the closure of this sensory phase of learning. If true, this could mean that the sensory period is shorter (at the very lower limit) than commonly believed in canaries and that PNN increase in lMAN could be a marker of and possibly contribute to the end of this sensitive period. Alternatively, plasticity in lMAN does not rely on changes in PNN expression. These alternative hypotheses should be tested in future work quantifying PNN in the brain of much younger canaries.

## Synthesis

Reviewing Editor: Darcy Kelley, Columbia University Decisions are customarily a result of the Reviewing Editor and the peer reviewers coming together and discussing their recommendations until a consensus is reached. When revisions are invited, a fact-based synthesis statement explaining their decision and outlining what is needed to prepare a revision will be listed below. The following reviewer(s) agreed to reveal their identity: Jon Sakata. Note: If this manuscript was transferred from JNeurosci and a decision was made to accept the manuscript without peer review, a brief statement to this effect will instead be what is listed below.

The authors analyzed the developmental formation of perineuronal nets (PNNs) in the song control system (SCS) of male canaries. Their study is comprehensive and offers interesting insights into the contribution of PNNs in the SCS to song development, learning, and performance. Because of their protracted period of song development, canaries could offer distinct insights into PNN function than the most extensively studied songbird, the zebra finch. The data are interesting and compelling, especially the findings that PNN expression peaks before canary songs are crystallized. The results of the T-implant part of the study will require further study to figure out what is going on (compared to prior studies) but the overall results for the main part of the paper are clear. Removing acronyms like PNN and spelling out the words would improve accessibility but otherwise the manuscript is exceptionally clearly written.

Major comments:

More information is needed to provide greater support for using canaries over zebra finches. The authors first mention the fact that the sensorimotor period in zebra finches is relatively short compared to canaries and overlaps with the sensory period. The authors mention that this makes inferring the function of PNNs difficult. Generally speaking, this angle should be toned down more because substantial inferences about PNNs can be based on fine grained analyses of PNN development in zebra finches (e.g., lines 102-104). In addition, it is important to communicate when the sensory period for vocal learning ends in canaries. Such information is tucked away in the Methods section, but it should be emphasized and discussed more extensively given the current “spin” of the manuscript. In addition, when writing about song development, this should consistently frame this in terms of the age of the birds (line 156-157, dph vs. season).

Predictions about PNN changes based on the closing of the sensory period vs. the end of sensorimotor development should be explicit. A summary figure at the end that synthesizes the findings would be useful; for example, a figure that depicts the timing of the sensory and sensorimotor periods and that includes information about PNN and PV neuron changes across this same time period. Such a visual (along with citations about the timing of the sensory vs. sensorimotor period) would help explain why the current results speak against a role of PNNs in closing the critical period for sensory learning.

A lot of brains were processed, and more information about the immunocytochemical runs (“batches”) is needed. Were all brains run in a single batch? If not, were groups balanced across batches? This is important because there can be differences in the intensity of staining across batches, and if groups are not balanced across batches, then group differences could simply reflect batch differences. Batch should also be included as a variable in the analysis. By removing variation that is not relevant to the experiment, adding this variable could increase statistical power, and could potentially explain away unexpected results (e.g., PNNs decreasing with testosterone).

While this paper is primarily about song performance and development and the history of the birds is complicated (e.g., birds were exposed to three different adult males over development), it would have been very useful to include information on the extent of song learning (similarity to tutors). Perhaps variation in PNN development could reflect the fidelity of song acquisition.

Based on the methods, data to make more definitive statements about the stability (crystallization) of song appear to be available. Birds were screened for 4 weeks before brains were collected, but only the songs produced during the last recording were analyzed. Song maturation is not only based on the stability of songs within a day but also on the stability of songs across days. So, at minimum, songs should be analyzed at two time points in order to make stronger statements about song maturation and stereotypy.

More information about song analysis is important. How Raven is analyzing the data is not clear (e.g., how are syllables segmented).

Minor comments:

Title: “Ontogenesis” is not used often in behavior. “ontogeny” is more appropriate (global change, e.g., line 109).

Line 38-39, “at the end of song learning during ontogeny” please be more specific whether discussing sensory or sensorimotor aspects of song learning. See also line 639.

Lines 46-48. This summary seems to contrast with the finding that PNNs “peak” before song crystallizes.

Line 61. “namely by preventing establishment of new synaptic connections” should be removed because this is conjecture and there are other potential mechanisms (e.g., strengthening existing connections, precision of PV neuron firing) that could explain vocal stability.

Line 73: “never change their song subsequently,”... “subsequent” to what?

Line 80: “analog” should be “analogous”

Line 104-line 107: this is useful information should be contextualized (i.e., why is this information useful).

Line 121: “3d” should be “3rd”

The developmental experiences of these birds is complicated. Were they effectively “tutored” by three males (or two males, if exclude father)?

Line 156-157. Would the end of the sensory period (according to this statement) be around 150-180 days of age? What is the primary literature detailing these time points?

Line 219: reword this sentence, since “evolution” is not appropriate here.

Line 276-277. What is the evidence that associates lower song entropy (as analyzed across all songs) with more stereotyped song? Should be explicit here and include a citation.

Lines 279-291. While qualitative assessments of song are useful, but may not be necessary here, given the large number of features computed. In addition, aspects like repetition and the ability to identify individual syllable types could be analyzed quantitatively. For example, for repetition, the difference in acoustic features between adjacent syllables could be computed to identify instances of pairs in which differences are very small (i.e., repeated syllables). With regard to syllable types, papers in zebra finches (e.g. Aronov et al., 2008) have analyzed the distribution of features and argued that the entropy (how peaked the distribution is) of the distribution changes as syllables become more well-formed. Papers from Ofer Tchernichovski's lab similarly describe song development in this manner, and it would be useful of the authors use similar metrics to describe song maturation in canaries.

Were song raters were blind to the age and endocrine state of the bird?

Line 338. Why was PNN and PV neuron expression in LMAN not analyzed?

General comment: The spring when birds are 55 dph vs. the spring when birds are adults (e.g., “spring after photostimulation”) should be differentiated. There are places in the manuscript where it's confusing (e.g., line 394).

Line 405-406: One bird was excluded from the analysis of song performance because it did not sing. This is reasonable. The bird could be included in the analysis of seasonal changes in song rate (number of songs per unit time), or in the analysis of PNNs and PV neurons. It's not clear whether this bird was excluded from all analyses.

Line 426: “evolution” should be replaced with “development”

Line 444: “improved” is a loaded term. Please replace.

Line 456-458: This sentence is confusing. If the lower quartiles increase, wouldn't that mean that power becomes distributed more toward the lower frequencies?

Line 482-483: typo “o fthe SCS”

Line 517-519: “older” PV neurons? How is the relative age of these neurons determined based on these stains?

Line 533: capitalize “x”

Line 536: “new” PNNs?

Line 538-540: Statistics should be separated by brain area. “The number of PNN and ... differed across time points in RA (________) and in Area X (________).” Overall, presentation of results could be cleaned up in this section.

Lines 587-589: Do sensory and sensorimotor period also overlap in canaries? (see lines 156-157)

Line 597: “terminates” is awkward . “The emergence of PNNs peaks during ...”

Line 647-649: Is the song fully mature at the time of the first spring? Can it not become more stereotyped across the first spring season? This is important to flesh out in order to make this statement.

Discussion: Iincreases in the motivation to sing (i.e., amount of time spent singing) happen before a number of changes in song performance. As such, the data demonstrate a dissociation between the motivation to sing (amount of song) and song performance. In the context of song development, this makes sense (since practice improves performance). But it would be useful to discuss this finding in the context of some of the authors' (and other researcher's) research on this dissociation.

General note on methods: It is important to point out that animals sampled in the spring, summer and autumn did not undergo any surgery for implantation, whereas all birds in the winter underwent a brief surgery for implants.

A recent paper out of Manfred Gahr's lab speaks to the role of testosterone in song crystallization in canaries and should be cited.
